# Unfolded protein response signaling impacts macrophage polarity to modulate breast cancer cell clearance and melanoma immune checkpoint therapy responsiveness

**DOI:** 10.18632/oncotarget.19849

**Published:** 2017-08-03

**Authors:** David R. Soto-Pantoja, Adam S. Wilson, Kenysha YJ. Clear, Brian Westwood, Pierre L. Triozzi, Katherine L. Cook

**Affiliations:** ^1^ Department of Surgery, Wake Forest School of Medicine, Winston-Salem, NC 27157, USA; ^2^ Department of Internal Medicine, Wake Forest School of Medicine, Winston-Salem, NC 27157, USA; ^3^ Comprehensive Cancer Center of Wake Forest Baptist Medical Center, Wake Forest School of Medicine, Winston-Salem, NC 27157, USA

**Keywords:** breast cancer, melanoma, immune therapy, ipilimumab, CTLA4

## Abstract

The unfolded protein response (UPR) is a stress pathway controlled by GRP78 to mediate IRE1, PERK, and ATF6 signaling. We show that targeting GRP78, IRE1, and PERK differentially regulates macrophage polarization. Specifically, PERK targeting enhanced macrophage proliferation and macrophage-mediated killing but not GRP78 or IRE1. Targeting UPR in cancer cells also differentially affected macrophage cytolytic capacity. Tumoral IRE1 or GRP78 inhibition enhanced macrophage-mediated cancer cell clearance. Conditioned media from GRP78-silenced cancer cells caused reciprocal regulation of CD80 and CD206, suggesting control of plasticity by secreted factors. GRP78 targeting in mice resulted in a cytokine shift and increased tumoral CD80+/CD68+ cells, suggesting an M1-like profile. Targeting UPR in both macrophage and cancer cells indicates that PERK or GRP78 reduction enhances macrophage clearance of cancer cells. Recent evidence suggests that macrophage polarization influences immune checkpoint therapy resistance. To determine whether UPR effects immunotherapy resistance, analysis of matched melanoma patient PBMC before/after developing ipilimumab resistance demonstrated increased UPR signaling and an M2-like macrophage population, supporting a novel role of UPR signaling and innate immune regulation in anti-CTLA-4 therapy resistance. These data suggest that targeting GRP78 or PERK promotes an anti-tumor immune response by either directly promoting macrophage cytolytic activity or indirectly by shifting tumoral cytokine secretion.

## INTRODUCTION

The unfolded protein response (UPR) is an endoplasmic reticulum (EnR) stress pathway activated when unfolded or misfolded proteins accumulate within the lumen of the EnR. When proteins accumulate within the EnR, the protein chaperone and master controller of UPR signaling, glucose-regulated protein 78 (GRP78; *HSPA5*), unbinds from the three UPR signaling arms allowing activation of inositol-requiring enzyme-1 (IRE1; *ERN1*), PKR-like endoplasmic reticulum kinase (PERK; *EIF2AK3*), and activating transcription factor 6 (*ATF6*). IRE1 stimulation leads to the unconventional splicing of x-box binding protein-1 (*XBP1*) to the highly active transcription factor XBP1-S (spliced). Stimulated IRE1 also has kinase activity; IRE1 phosphorylates c-Jun N-terminal kinase (JNK), which may lead to apoptosis with pro-longed UPR stimulation [1]. ATF6, once unbound from GRP78, translocate to the Golgi complex where it is cleaved by site1/site2 proteases to form the active transcription factor. ATF6 promotes the transcription of protein chaperones (GRP78 and GRP94) and un-spliced XBP1, feeding-back into UPR signaling pathway. PERK activation leads to a halt of cap-dependent protein translation through phosphorylation of eIF2α and promotion of ATF4 transcription. Activation of PERK halts cap-dependent protein translation, thereby lessening the unfolded protein load in the EnR. If not resolved in a timely manner, PERK activation can lead to apoptosis through ATF4-mediated C/EBP homology protein (CHOP; *DDIT3*) induction. Therefore, in normal cells, UPR activation is pro-survival; however, extended UPR signaling promotes apoptosis [2–4].

UPR signaling is upregulated in many different types of cancers, including breast cancer [5–7] and melanoma [8], and is associated with the development of therapeutic resistance [9–12]. These data suggest the importance of targeting UPR signaling as a possible cancer therapy. In fact, there are several ongoing clinical trials in the United States investigating GRP78, PERK, and XBP1-targeted therapeutics for cancer as well as for rheumatoid arthritis and type 2 diabetes (clinicaltrials.gov). We previously showed that GRP78 is upregulated in human breast tumors and leads to endocrine therapy resistance [9]. We recently showed that inhibiting GRP78 in human orthotopic xenografts potentiates tamoxifen therapy effectiveness in sensitive tumors and restores endocrine therapy responsiveness in resistant tumors [13]. We demonstrated that targeting GRP78 led to regulation of lipid metabolism resulting in elevated cytosolic concentrations of polyunsaturated fatty acid metabolites [13]. In these GRP78-inhibited tumors, the CD68 positive macrophage population was significantly increased, suggesting that targeting UPR signaling has critical effects on the tumor microenvironment [13]. Therefore, consideration of each UPR signaling component and how it effects the different cellular compartments of the tumor microenvironment needs to be investigated to optimally induce an antitumor immune effect and inhibit tumor epithelial cell growth.

While recent data highlights the importance of UPR signaling in macrophages in the development of atherosclerosis [14–17], the specific role of each UPR signaling component in regulating innate immunity and macrophage polarity in cancer is unknown. Macrophage polarity can be defined as either M1 classical activated or M2 alternatively activated. M1 macrophages are pro-inflammatory while M2 macrophages are anti-inflammatory. Tumor- associated macrophages are more M2-like, promoting angiogenesis, tumor immunosuppression, and metastatic spread [18]. Highly secretory cell types, such as immune cells, have large EnR cell compartments and elevated UPR components to accommodate the increased protein synthesis/folding required by these cell types. Therefore, these cell types may be highly sensitive to EnR stress.

We now show that UPR signaling regulates innate antitumor immune responses. Targeting PERK directly promotes M1-like macrophage cytolytic activity and clearance of tumor cells while targeting GRP78 indirectly promotes M1-like macrophages by shifting tumoral cytokine secretion. Recently, resistance to immune-checkpoint therapy was attributed to macrophage polarization [19]. Immunosuppressive macrophages may release cytokines that regulate genes involved in antigen presentation and immune activation affecting T cell function. Therefore, lack of responses to immune checkpoint inhibitor therapy may be regulated by UPR signaling to promote a tumor M2-like macrophage population that is associated with disease progression. Our results show that inhibition of PERK stimulates macrophage proliferation and enhanced macrophage cytolytic clearance of breast cancer cells when compared to control transfected cells. Knockdown of PERK by RNAi increased iNOS and inhibited Arg-1 protein expression, suggesting an M1-like macrophage phenotype.

Inhibition of UPR signaling components in breast cancer epithelial cells can also indirectly affect macrophage polarity. Knockdown of GRP78 or IRE1 in tumor epithelial cells increases macrophage cytolytic activity when compared with control or PERK siRNA transfected breast cancer cells. Moreover, treatment with conditioned media from breast cancer cells transfected with control or GRP78 siRNA demonstrated that secreted factors induced by GRP78 targeting in breast cancer cells enhances M1-like macrophage polarity. Moreover, in melanoma patients undergoing anti-CTLA4 immunotherapy, circulating PBMC after loss of therapeutic effectiveness had elevated UPR signaling and increased M2-like macrophage population when compared with patient matched PBMC from before therapy. These data suggest that ipilimumab may induce UPR signaling to promote a tumor-associated M2-like macrophage population that is associated with disease progression. These data suggest the importance of investigating drug target effects in both tumor epithelial cells and immune cells to maximize therapeutic effectiveness.

## RESULTS

### Chemical UPR inducing agents differentially affect macrophage polarity

**Figure 1:**
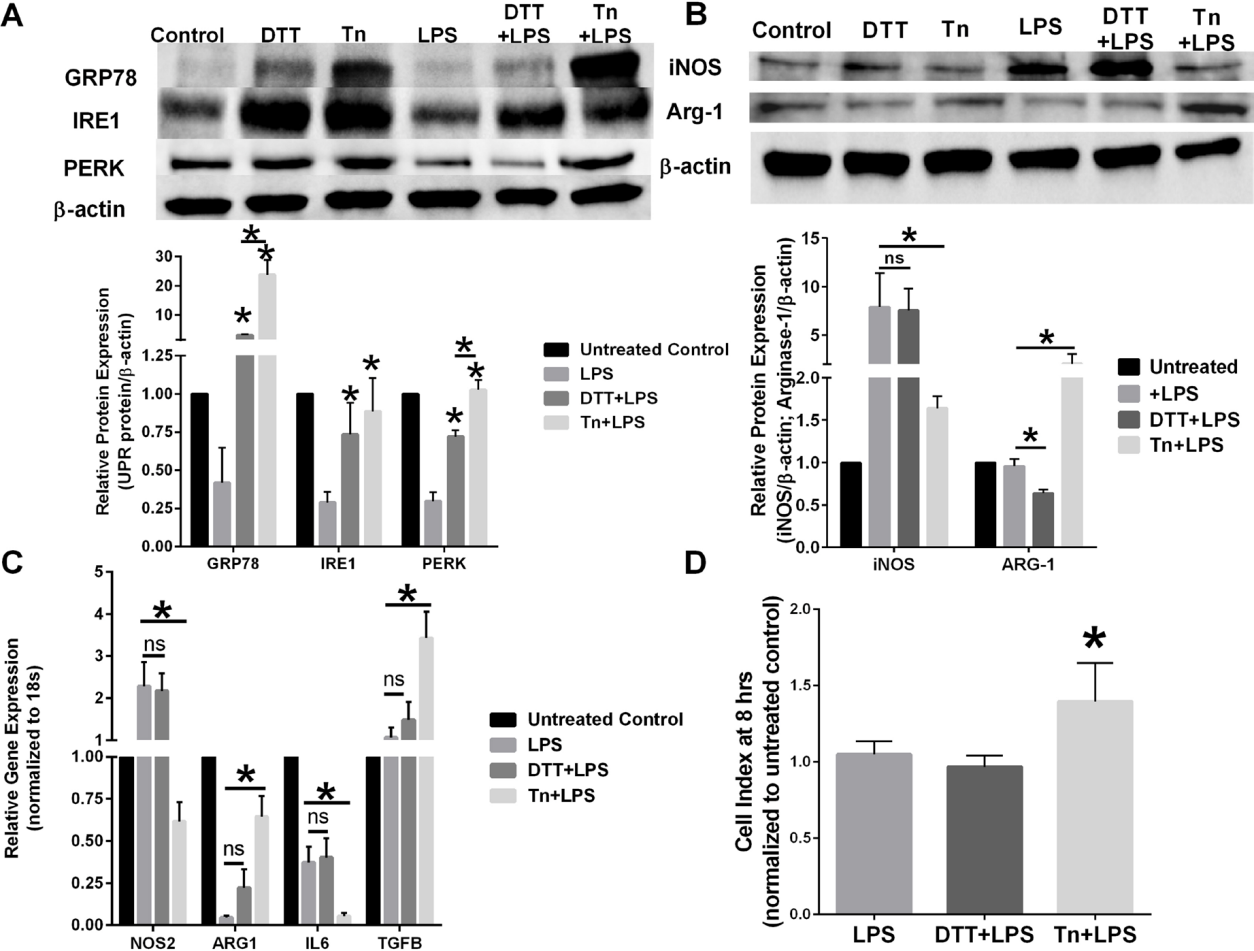
Chemical UPR stressor agents differentially effects macrophage polarity (**A**) RAW 264.7 cells were pretreated with vehicle, 1 mM DTT, or 1 μg/mL tunicamycin (Tn) for 4 hours before treatment with 1 μg/mL LPS for 24 hours. GRP78, IRE1, and PERK were measured by Western blot hybridization. Protein loading was normalized to β-actin. *n* = 3; *p < 0.05. (**B**) RAW 264.7 cells were pretreated with vehicle, 1 mM DTT, or 1 μg/mL tunicamycin (Tn) for 4 hours before treated with 1 μg/mL LPS for 24 hours. iNOS and Arg-1 were measured by Western blot hybridization. Protein loading was normalized to β-actin. *n* = 3; *p < 0.05. (**C**) RT-PCR of iNOS, ARG-1, IL-6, IL-10, and IL-12 in RAW 264.7 cells pretreated with vehicle, 1 mM DTT, or 1 μg/mL tunicamycin (Tn) for 4 hours before treatment with 1 μg/mL LPS for 24 hours. Gene expression was normalized to the 18s housekeeping gene. *n* = 5; *p < 0.05. (**D**) 4T1 breast cancer cells were plated in an ACEA E-plate for 24 hours; RAW 264.7 macrophages pretreated with vehicle, 1 mM DTT, or 1 μg/mL Tn for 24 hours were then added to the E-plate. Each well was treated with 1 μg/mL LPS and the cell index was measured at 8 hours by electrical impedance. *n* = 3; *p < 0.05.

RAW 264.7 (mouse macrophage cell line) were pre-treated with 0.1% ethanol vehicle control, 1 mM DTT, or 1 μg/mL tunicamycin (Tn) for 4 hours before stimulation with 1 μg/mL LPS for 24 hours. Induction of UPR signaling was confirmed by Western blot hybridization (Figure 1A). Both chemical EnR stress inducing agents activated UPR signaling as measured by GRP78. Interestingly, LPS activation of macrophages decreased UPR signaling protein expression. Pre-treatment with Tn restored PERK expression to that of the untreated control. Western blot analysis of macrophage protein lysates demonstrated that pretreatment with Tn, but not DTT, prevented LPS-mediated iNOS induction (Figure 1B). Furthermore, pretreatment with DTT significantly reduced the M2-like macrophage protein marker Arg-1 when compared with LPS-treated RAW 264.7 cells, suggesting reciprocal regulation of macrophage polarity by differing EnR stress inducing chemical agents. Pre-treatment of RAW 264.7 cells with EnR stress inducing agents also differentially affected gene expression of M1/M2 macrophage markers (Figure 1C). Tn pretreatment reduced LPS-mediated stimulation of iNOS and IL6 gene expression when compared with LPS stimulation alone. Furthermore, Arg-1 and TGF-β gene expression were significantly upregulated in Tn pre-treated macrophages when compared with LPS-stimulated macrophage gene expression. Chemical induction of EnR stress by either DTT or Tn also affected macrophage cytolytic activity. Pretreatment of RAW 264.7 cells with Tn significantly reduced macrophage-mediated clearance of breast cancer cells when compared with DTT pretreatment or LPS-only treated macrophage cytolytic capacity (Figure 1D).

### Inhibiting each UPR signaling component by RNAi differentially affects macrophage activity

**Figure 2:**
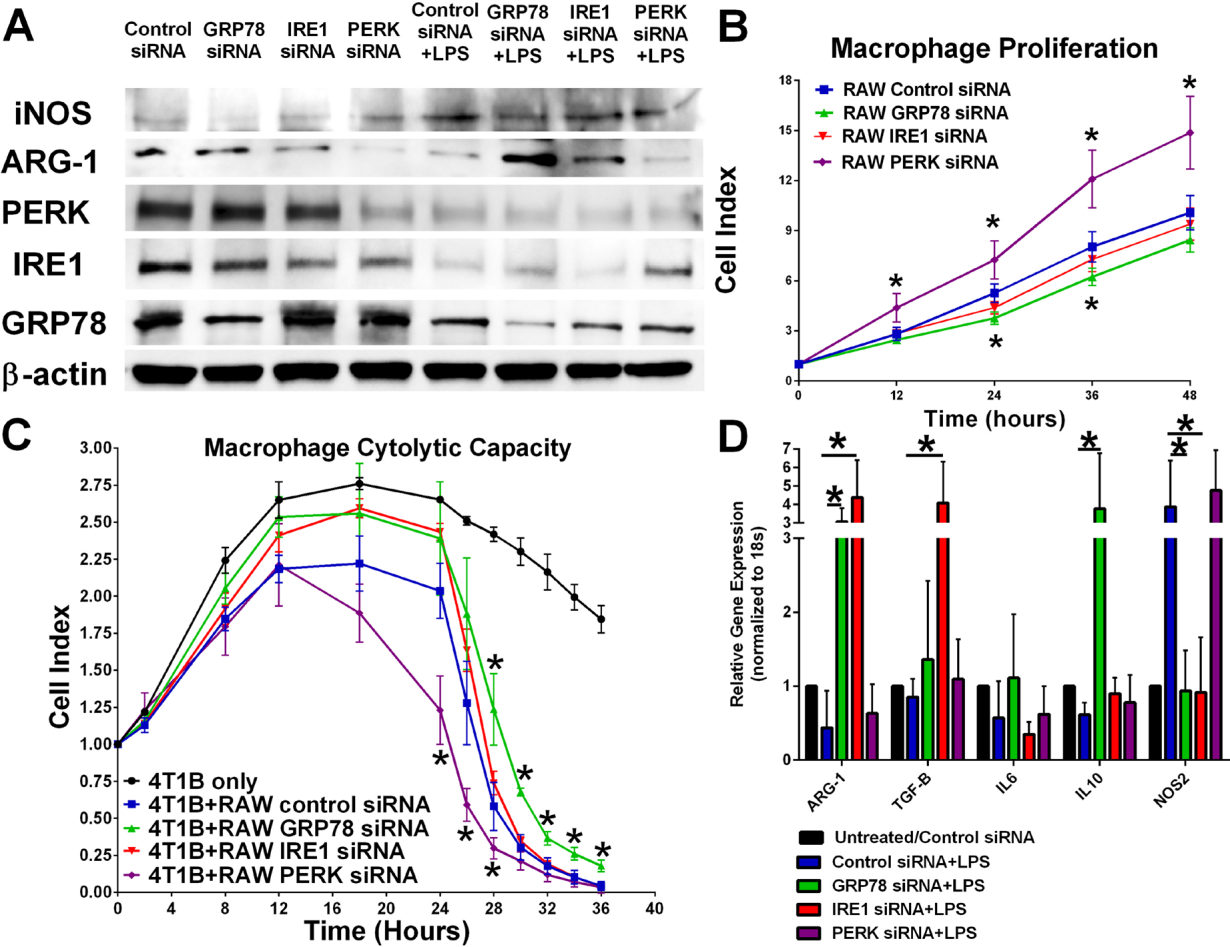
Knockdown of various UPR components differentially effects macrophage proliferation and plasticity (**A**) UPR signaling components IRE1, PERK, and GRP78 in RAW 264.7 cells were inhibited by RNAi treatment for 24 hours followed by treatment with 1 μg/mL LPS for 24 hours. iNOS, Arg-1, PERK, IRE1, and GRP78 were measured by Western blot hybridization. Protein loading was normalized to β-actin. (**B**) UPR signaling components IRE1, PERK, and GRP78 were inhibited by RNAi for 24 hours and then plated in an ACEA E-plate. Each well was treated with 1 μg/mL LPS and the cell index was measured every 12 hours by electrical impedance. *n* = 3; *p < 0.05. (**C**) 4T1B breast cancer cells were plated in an ACEA E-plate for 24 hours; then 5 × 10^4^control, IRE1, PERK, or GRP78 siRNA transfected RAW 264.7 macrophages were added to the E-plate. Each well was treated with 1 μg/mL LPS and the cell index was measured every 4 hours by electrical impedance. *n* = 3; *p < 0.05. (**D**) RT-PCR analysis of iNOS, ARG-1, IL-6, IL-10, and IL-12 gene expression in control, IRE1, PERK, or GRP78 siRNA transfected RAW 264.7 cells treated with 1 μg/mL LPS for 24 hours. Gene expression was normalized to 18S housekeeping gene. *n* = 4; *p < 0.05.

Mouse macrophage RAW 264.7 cells were transfected with control, GRP78, IRE1, or PERK siRNA for 24 hours before stimulation with 1 μg/mL LPS for 24 hours. Western blot analysis of macrophage protein lysates indicate that knockdown of PERK increased LPS-mediated iNOS induction and reduced Arg-1 protein expression. There was a modest increase in Arg-1 protein levels in GRP78 and IRE1 silenced macrophage cells when compared with LPS-stimulated alone (Figure 2A). Interestingly, LPS stimulation alone resulted in decreased GRP78 and PERK protein levels indicating a key role of UPR signaling in macrophage activation. Inhibition of PERK signaling increased RAW 264.7 macrophage proliferation while GRP78 inhibition modestly reduced macrophage proliferation when compared with control or IRE1-transfected macrophage cells (Figure 2B). Knockdown of UPR signaling components by RNAi affected macrophage cytolytic capacity (Figure 2C). Specifically, targeting PERK signaling increased macrophage-mediated clearance of breast cancer cells while reducing GRP78 in the macrophages decreased cytolytic activity. Transfection of RAW 264.7 cells with UPR targeting siRNA also differentially affected gene expression of M1/M2 macrophage markers (Figure 2D). GRP78 and IRE1 knockdown reduced LPS-mediated stimulation of iNOS while significantly increasing Arg-1 expression. IRE1 knockdown alone significantly elevated macrophage TGF-β expression, while GRP78 knockdown alone significantly elevated IL-10 expression. No significant differences in gene expression were observed in control siRNA+LPS and PERK siRNA+LPS treatments.

### Targeting UPR signaling components affects cellular bio-energetics

**Figure 3:**
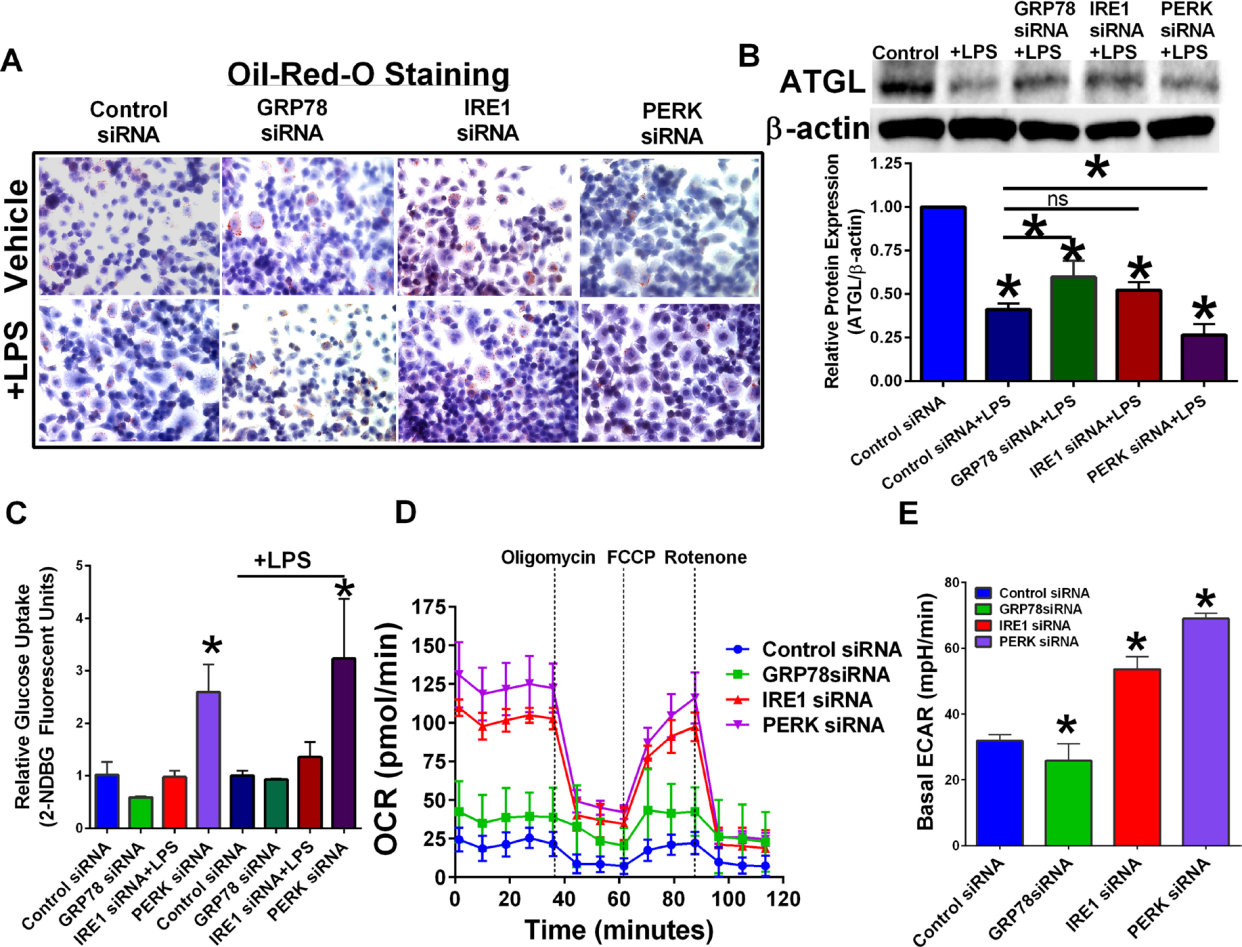
Targeting UPR signaling components differentially regulate cellular metabolism (**A**) RAW 264.7 macrophage cells were transfected with control, GRP78, IRE1, or PERK siRNA for 24 hours, and then treated with or without LPS. Intracellular lipids were stained for oil-red-o and representative images obtained at 40×. (**B**) UPR signaling components IRE1, PERK, and GRP78 in RAW 264.7 cells were inhibited by RNAi treatment for 24 hours followed by treatment with 1 μg/mL LPS for 24 hours. ATGL was measured by Western blot hybridization. Protein loading was normalized to β-actin. (**C**) Glucose uptake in RAW 264.7 cells transfected with scrambled control or UPR targeting siRNA for 24 hours. *n* = 3; *p < 0.05. (**D**) Mitochondrial metabolomics was determined in transfected RAW 264.7 macrophages using a Seahorse Bioanalyzer. (**E**) Basal ECAR rates in transfected RAW 264.7 macrophage cells. *n* = 3–4; *p < 0.05.

RAW 264.7 cells were transfected with control, GRP78, IRE1, or PERK siRNA. Knockdown of GRP78 increased the overall lipid content of macrophages (Figure 3A) as shown by an increase in oil-red-o staining. Adipose triglyceride lipase (ATGL) cleaves triacylglycerol to generate non-esterified fatty acids and is found in most tissues of the body. Free fatty acid metabolites generated by ATGL can be used in mitochondrial β-oxidation as an energy source [20]. β-oxidation promotes an M2-like macrophage phenotype [21]. Treatment with LPS reduced ATGL protein levels regardless of UPR inhibition; however, GRP78 targeting +LPS treated macrophages had elevated ATGL when compared to ATGL protein levels in control transfected + LPS macrophages (Figure 3B). Targeting PERK potentiated LPS-mediated ATGL reduction. Transfected RAW 264.7 cells were treated with 2-NDGB to determine effect of UPR targeting on glucose uptake (Figure 3C). Targeting PERK significantly elevated macrophage glucose uptake regardless of LPS stimulation. A Seahorse bioanalyzer was used to determine UPR targeting effects on mitochondrial metabolism (Figure 3D). Inhibition of IRE1 or PERK led to an elevated oxygen consumption rate (OCR). Reduced OCR was associated with a reduction in glycolysis and elevated β-oxidation, suggesting an M2-like phenotype in unstimulated RAW 264.7 and GRP78-targeted RAW 264.7 macrophages. Basal ECR was increased in IRE1 and PERK targeted macrophages while GRP78 knockdown had reduced basal ECR (Figure 3E).

### Primary bone marrow-derived macrophage from wild-type and GRP78 heterozygous mice display contradistinctive plasticity

**Figure 4:**
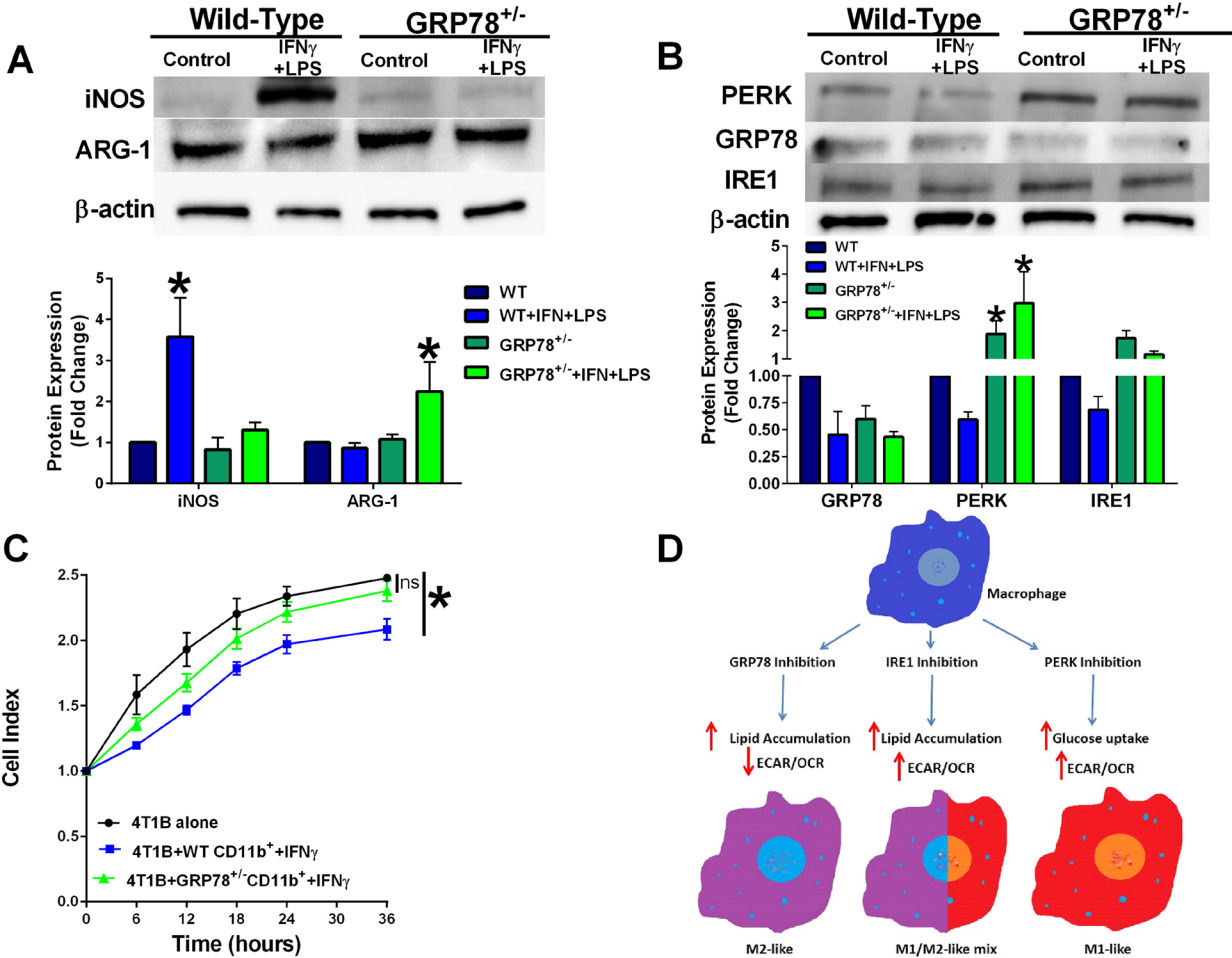
Macrophages isolated from GRP78 heterozygous mice have reduced cytolytic capacity (**A**) Protein lysates from IFNγ-treated bone marrow cells from wild-type and GRP78 heterozygous mice were analyzed for iNOS, ARG-1, and β-actin by Western blot hybridization. *n* = 3; *p < 0.05. (**B**) Protein lysates from IFNγ-treated bone marrow cells from wild-type and GRP78 heterozygous mice were analyzed for PERK, GRP78, IRE1, and β-actin by Western blot hybridization. *n* = 3; *p < 0.05. (**C**) 4T1B breast cancer cells were plated in an ACEA E-plate for 24 hours and then wild-type or GRP78 heterozygous CD11b+ cells that were pre-treated with IFNγ for 48 hours + LPS were added to the E-plate. Cell index was measured every 6 hours by electrical impedance. *n* = 3; *p < 0.05. (**D**) Signaling schematic representing how UPR targeting differentially regulates cellular bioenergetics to control macrophage polarity.

CD11b+ cells were isolated from bone marrow of wild-type and GRP78 heterozygous mice and treated with IFNγ for 48 hours and then stimulated with LPS for 24 hours. Heterozygous GRP78 expression prevented LPS-stimulated iNOS protein induction suggesting a possible shift in macrophage polarity (Figure 4A). Bone marrow-derived CD11b+ cells from heterozygous GRP78 mice also displayed increased PERK protein expression (Figure 4B). GRP78 heterozygousity also affected IFNγ-stimulated CD11b+ cell-mediated 4T1B breast cancer cell clearance as measured by electrical impedance (Figure 4C), providing further evidence that GRP78 modulates macrophage function. A signaling schematic of how UPR targeting in the macrophage modulates metabolism to differentially regulate macrophage polarity is shown in Figure 4D.

### Targeting UPR signaling components in breast cancer cells differentially regulates macrophage polarity

**Figure 5:**
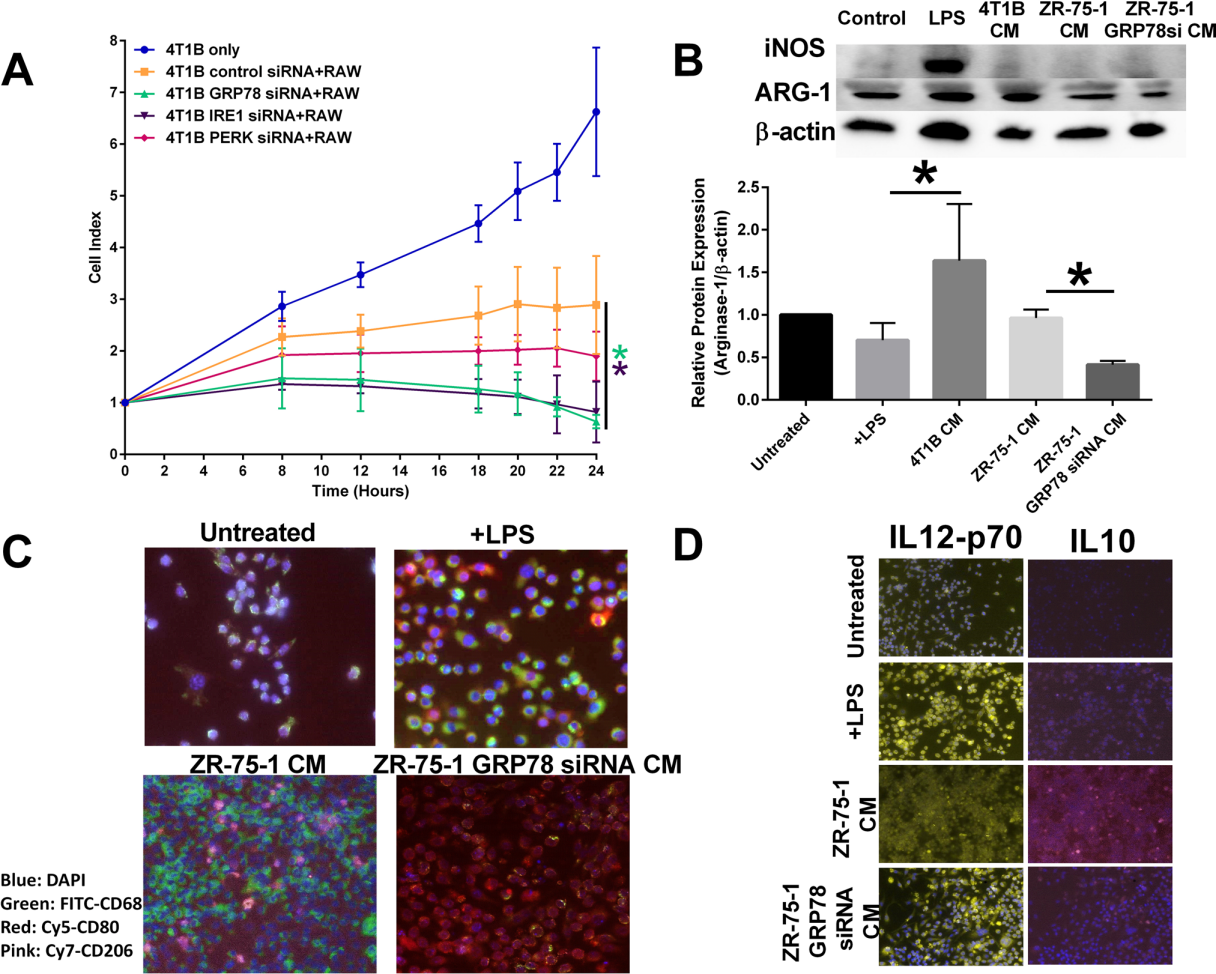
Inhibiting UPR signaling components in the tumor epithelial cell affects macrophage polarity (**A**) Control, IRE1, PERK, or GRP78 transfected 4T1B breast cancer cells were plated in an ACEA E-plate and RAW 264.7 macrophages were added to the E-plate. Each well was treated with 1 μg/mL LPS and the cell index was measured by electrical impedance. *n* = 3; *p < 0.05. (**B**) Conditioned media from control or GRP78 siRNA transfected 4T1B or ZR-75-1 breast cancer cells were used to treat RAW 264.7 cells for 24 hours. iNOS and Arg-1 were measured by Western blot hybridization. Protein loading was normalized to β-actin. *n* = 3; *p < 0.05. (**C**) Vehicle treated, LPS treated, ZR-75-1 conditioned media (CM), or GRP78-silenced ZR-75-1 conditioned media were used to treat RAW 264.7 macrophage cells for 24 hours. Macrophages were stained for CD68-FITC (green), CD80-Cy5 (red), or CD206-cy7 (pink) and counterstained with DAPI. The M1/M2-like macrophage population was determined by immunocytochemistry. (**D**) Vehicle treated, LPS treated, ZR-75-1 conditioned media, or GRP78-silenced ZR-75-1 conditioned media were used to treat RAW 264.7 macrophage cells for 24 hours. Macrophages were stained for IL12-Cy3 (yellow) or IL10-cy7 (pink) and counterstained with DAPI. The M1/M2-like macrophage population was determined by immunocytochemistry.

Inhibition of the various UPR components in 4T1B breast cancer cells also affected untransfected RAW 264.7 cytolytic capacity. Inhibition of GRP78 or IRE1 in breast cancer cells led to an increased macrophage cytolytic capacity as measured by cell impedance. PERK inhibition in breast cancer cells had no overall effect on macrophage activity (Figure 5A). RAW 264.7 cells were exposed to conditioned media from 4T1B breast cancer cells or control transfected or GRP78-silenced ZR-75-1 breast cancer cells. Western blot hybridization demonstrated that conditioned media from GRP78 inhibition of breast cancer cells reduced the M2 marker, Arg-1 (Figure 5B). Moreover, conditioned media from GRP78-silenced breast cancer cells increased macrophage CD80+ expression (an M1-like macrophage marker) while control transfected ZR-75-1 conditioned media showed elevated macrophage CD206+ an M2-like macrophage marker) cells (Figure 5C). Furthermore, GRP78-silenced breast cancer conditioned media increased macrophage IL-12 when compared to control breast cancer conditioned media, suggesting that GRP78-regulated secreted factors modulated macrophage polarity (Figure 5D).

### Dual targeting of UPR signaling components in both target and effector cells regulates macrophage activity

**Figure 6:**
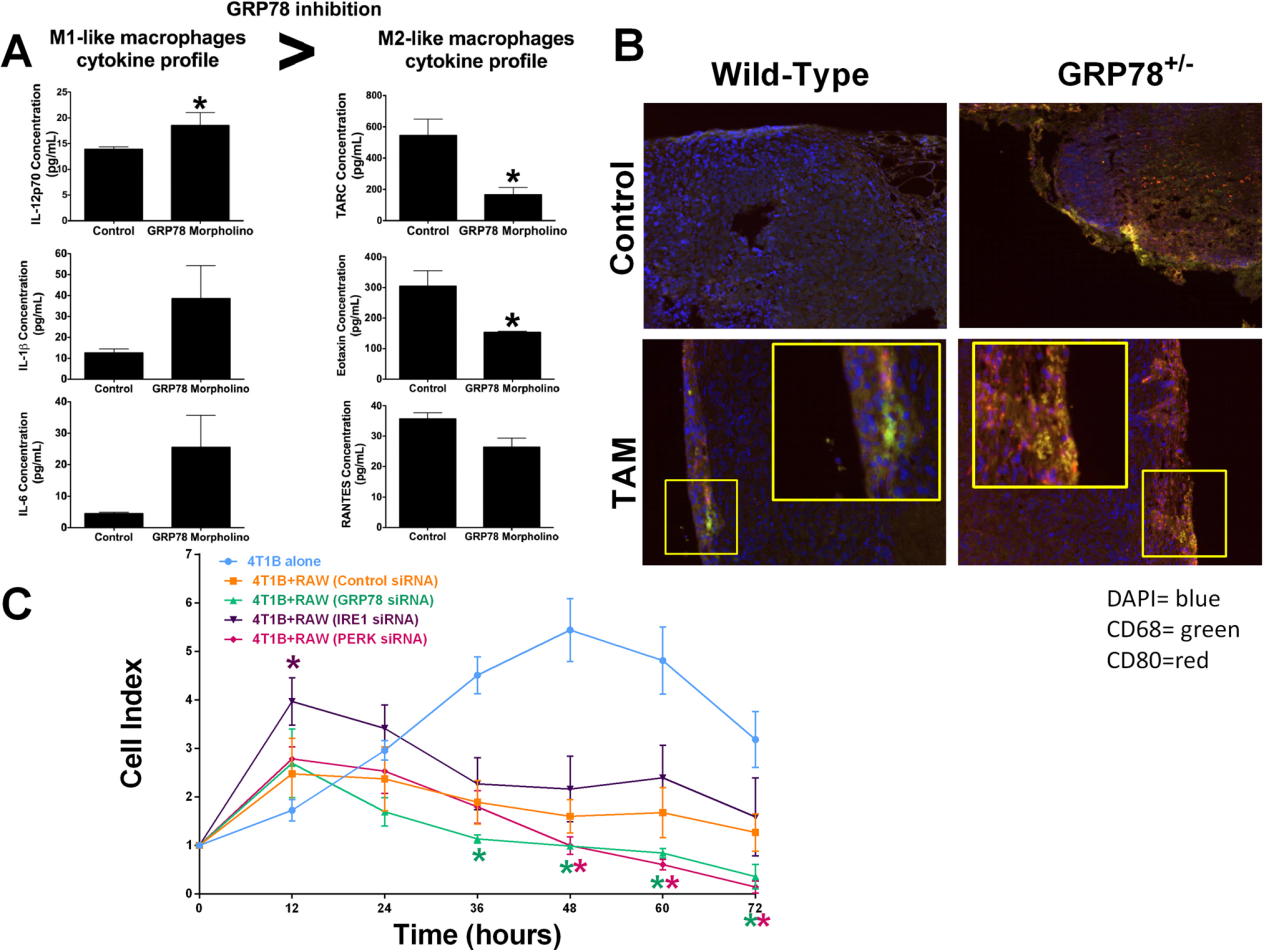
Dual targeting of UPR signaling in both tumor epithelial and macrophage cells affects macrophage recruitment and activity (**A**) IL-12p70, IL-1β, IL-6, TARC, Eotaxin, and RANTES were measured by ELISA from serum of WT and GRP78 morpholino treated mice. *n* = 4; *p < 0.05. (**B**) DMBA-induced mammary tumors from WT and GRP78 heterozygous mice (untreated or treated with tamoxifen) were stained with fluorescently labelled CD68 (green) or CD80 (red). Tumor sections were counterstained with DAPI. (**C**) Control, IRE1, PERK, or GRP78 transfected 4T1B breast cancer cells were plated in an ACEA E-plate and then control, IRE1, PERK, or GRP78 transfected RAW 264.7 macrophages were added to the E-plate. Each well was treated with 1 μg/mL LPS and the cell index was measured every 12 hours by electrical impedance. *n* = 3; *p < 0.05.

Systemic inhibition of GRP78 in BALB/c mice using a mouse specific GRP78-targeting morpholino increased circulating IL-12p70 cytokine concentrations. There was also a trend for GRP78-inhibition to increase circulating serum IL-1β and IL-6 concentrations. Circulating TARC (CCL17), eotaxin (CCL11), and RANTES (CCL5) were decreased in mice with reduced GRP78, suggesting that inhibiting GRP78 promotes a cytokine shift favoring M1-like macrophages (Figure 6A). Mammary carcinogenesis was induced by DMBA in wild-type and GRP78 heterozygous mice as previously described [22]. Wild-type and GRP78 heterozygous tumor-bearing mice were treated with tamoxifen and fixed tumors were stained for CD68 and CD80. GRP78 heterozygous mice displayed increased CD68/CD80 co-localization when compared with wild-type tumors, suggesting increased M1-like macrophages infiltrated the tumors (Figure 6B). Inhibition of UPR signaling components in both breast cancer cells and macrophage cells simulates systemic therapy treatment options which would be observed in patients. GRP78, PERK, or IRE1 UPR signaling arms were inhibited by RNAi in both 4T1B breast cancer cells and RAW 264.7 macrophage cells. Macrophage cytolytic capacity was measured by cell impedance. Dual inhibition of either PERK or GRP78 enhanced macrophage-mediated clearance of breast cancer cells, when compared with control or IRE1 inhibition (Figure 6C).

### Ipilimumab therapy in human melanoma patients affects UPR signaling and modulates macrophage polarity in circulating PBMC

**Figure 7:**
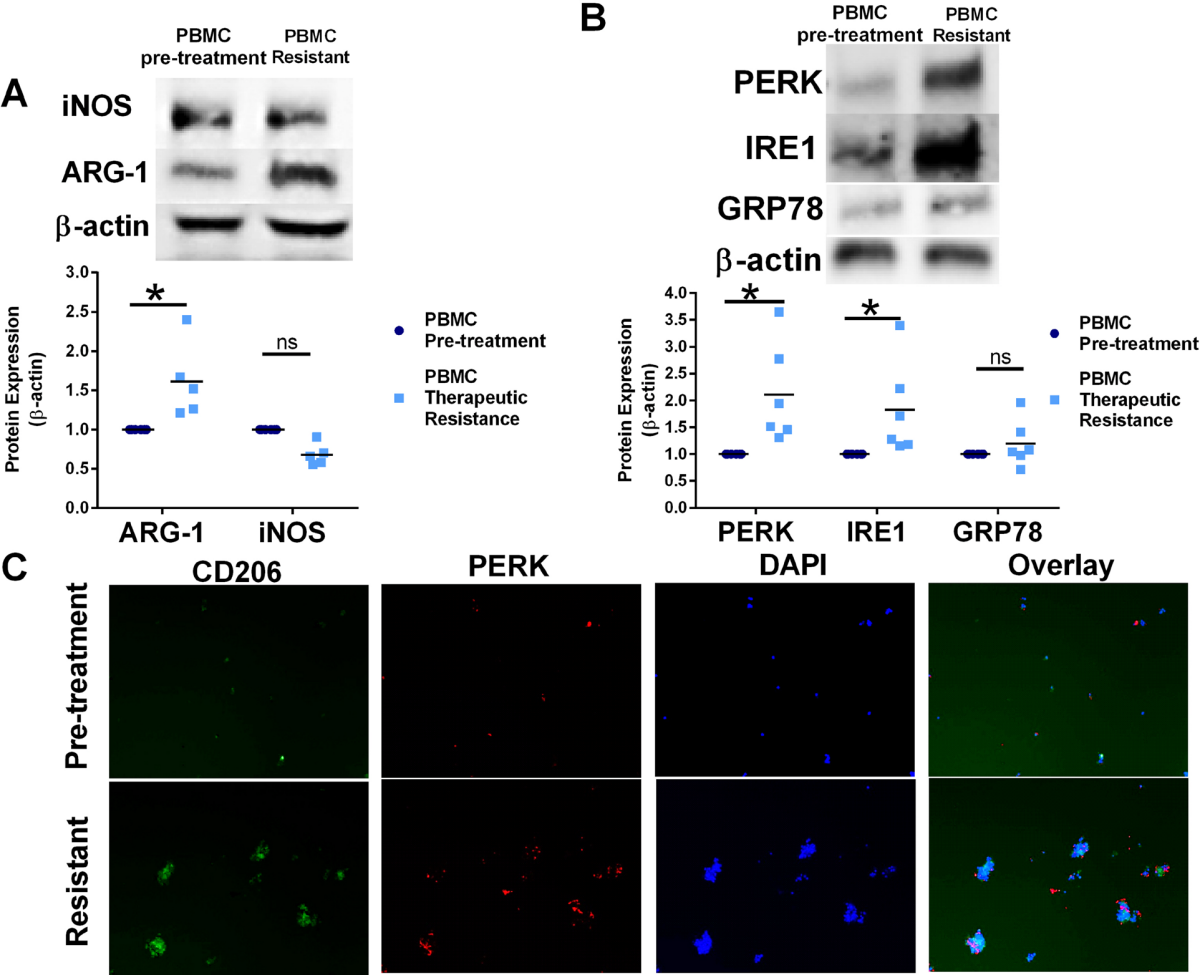
Resistance to CTLA-4 targeting immunotherapy modulates UPR signaling and shift macrophage polarity in human PBMC (**A**) Western blot analysis of Arg-1 and iNOS protein expression from matched human PBMC isolated from melanoma patients before Ipilimumab treatment or after progression of disease (Ipilimumab resistance). *n* = 5 *p = 0.02. (**B**) Western blot analysis of PERK, GRP78, and IRE1 protein expression from matched human PBMC isolated from melanoma patients before Ipilimumab treatment or after progression of disease (Ipilimumab resistance). *n* = 6; *p = 0.003 (**C**) Adherent PBMC from matched human PBMC isolated from melanoma patients before treatment and after ipilimumab therapy resistance were stained with CD206 M2-like macrophage marker and PERK antibodies. Cells were counterstained with DAPI.

Circulating PBMC were isolated from patients before ipilimumab therapy or after disease progression, modeling ipilimumab therapy resistance. Western blot analysis of protein lysates from matched patient adherent PBMC populations demonstrated elevated Arg-1 protein expression in adherent PBMC after the loss of ipilimumab treatment effectiveness, suggesting an increased pro-tumorigenic circulating M2-like macrophage population (Figure 7A). Western blot analysis of protein lysates from patient PBMC also indicates increased PERK and IRE1 protein expression in PMBC after the development of ipilimumab resistance when compared to PBMC protein lysates before treatment (Figure 7B). In support of these data, patient adherent PBMC populations after CTLA-4 targeted therapy had increased CD206+ cells and PERK+ cells (Figure 7C). These data suggest significant induction of UPR signaling and modulation macrophage plasticity after the development of resistance to ipilimumab therapy and disease progression.

## DISCUSSION

The EnR stress pathway, the UPR, is under investigation as a possible cancer therapeutic target. UPR signaling components are upregulated in many different types of cancers, including breast cancer and melanoma [5, 6, 8, 9]. Upregulation of UPR signaling components often leads to cancer cell survival and is correlated with therapeutic agent resistance [9–11, 23, 24], highlighting the importance of targeting this pathway. While inhibition of UPR signaling is being explored as a cancer cell therapeutic, the effect of targeting UPR signaling in the tumor microenvironment is unknown.

Using chemical agents such as DTT or Tn to induce EnR stress, we show that these agents have differing effects on macrophage function. DTT is a strong reducing agent and prevents the formation of disulfide bonds leading to the activation of UPR signaling. Tn is an inhibitor of the UDP-N-acetylglucosamine-dolichol phosphate N-acetylglucosamine-1-phosphate transferase (GPT) that blocks glycoprotein synthesis stimulating UPR signaling [25]. Both DTT and Tn stimulate UPR signaling in macrophage cells, albeit with differing potencies; Tn significantly elevated GRP78 and PERK when compared with DTT pretreated cells (Figure 1A). However, unlike DTT, pre-treatment with Tn prevents LPS-mediated M1-like stimulation of RAW 264.7 mouse macrophage cells (Figure 1B and 1C) and prevented macrophage-mediated endocytosis of breast cancer cells (Figure 1D). Previous reports showed that DTT and ER chaperones stabilize paraoxonase 2 (PON2) that may promote a more M2-like phenotype in atherosclerosis [26, 27], which was not observed in the current study. Whether these differential effects on macrophage plasticity observed by chemical inducers of UPR are due to the differing potency of UPR stimulation (Figure 1A), the possible requirement of glycosylated protein biosynthesis for macrophage stimulation [28] or the highly reductive basal redox state existing in macrophages that may curtail DTT efficacy are still unclear [29].

Interestingly, LPS treatment which promotes an M1-like macrophage phenotype reduced GRP78 and PERK protein levels (Figure 2A), suggesting that decreased UPR signaling may promote M1 macrophage polarity. Previous reports in the literature support a critical role of UPR in macrophage differentiation, where elevated UPR signaling was associated with M2 macrophage polarity and foam cell formation via JNK and PPARγ-dependent pathways [30]. Due to the lack of specificity from chemical agents triggering UPR signaling, we knocked out each UPR signaling arm individually (IRE1, GRP78, and PERK) to determine the effect of UPR inhibition on macrophage plasticity. Our results show that targeting the various UPR signaling arms through RNAi has differential effects on macrophage polarity (Figure 2). Inhibition of PERK increased M1-like gene expression and the protein marker iNOS (Figure 2A and 2D), macrophage proliferation (Figure 2B), and macrophage-mediated killing of breast cancer cells (Figure 2C). PERK activation inhibits cap-dependent protein translation, which includes many of the M1-like pro-inflammatory cytokines [31], suggesting that elevated PERK signaling may reduce macrophage activity. Furthermore, prolonged PERK activity promotes macrophage apoptosis through induction of CHOP signaling [32], supporting our data demonstrating an increased cell index in PERK inhibited macrophage (Figure 2B). Taken together, these data suggest that targeting PERK would enhance an anti-tumor innate immune response. IRE1 inhibition had no overall significant effect on macrophage polarity (Figure 2A and 2D), proliferation (Figure 2B), or cytolytic activity (Figure 2C). IRE1 activation leads to the unconventional splicing of XBP1, to form the highly active transcription factor XBP1-S [3]. Previous reports showed that XBP1 is critical for TLR activation of cytokine production [33]. Since we observed no significant changes in IL-6 transcription in IRE1 knockdown macrophages (Figure 2D) and no overall effect on macrophage-mediated breast cancer cell killing (Figure 2C), our data suggest that these effects may be independent of TLR activation. Other reports indicate that elevated levels of XBP1 in tumor-associated dendritic cells disrupt anti-tumor T-cell immunity through increased lipid peroxidation and lipid accumulation. Dendritic cell specific XBP1 deletion restored anti-tumor immune function in ovarian cancer, suggesting an immune benefit of targeting IRE1 in this immune cell subtype [34]. Interestingly, inhibiting GRP78 resulted in a more M2-like phenotype (Figure 2A and 2D), with decreased macrophage proliferation (Figure 2B), and a decreased macrophage-mediated breast cancer cell killing (Figure 2C). GRP78 inhibition often leads to activation of UPR signaling [3]. We observed increased PERK and IRE1 protein levels in GRP78 inhibited macrophages (Figure 2A), suggesting that elevated UPR signaling promotes an M2 macrophage response, which may have negative implications in cancer therapy efficacy. On the other hand, these data suggest that GRP78 targeting drugs may benefit atherosclerosis patients.

To further investigate the molecular mechanism of how GRP78 targeting in macrophages promoted a M2-like phenotype, we determined the effect of UPR targeting on cellular energetics and lipid metabolism. We previously showed that targeting GRP78 in breast cancer cells elevated the total lipid content [13]. Other studies show that M1 and M2-like macrophage polarity relies on different cellular energetic pathways; M1-macrophages undergo glycolysis while M2-macrophages perform β-oxidation of free fatty acid metabolites [21]. We now show that inhibition of GRP78 and IRE1 elevated the lipid content of macrophages (Figure 3A). Addition of LPS to activate RAW 264.7 macrophages decreased ATGL protein expression. Since macrophages are dependent on ATGL activity for the generation of free fatty acids to use as substrates for mitochondrial β-oxidation [35, 36], our data suggest that reduction of ATGL may promote M1-like macrophage polarity. Targeting GRP78 reduced LPS-mediated ATGL reduction, while PERK knockdown enhanced LPS-induced ATGL protein inhibition, supporting our data showing that GRP78 targeting in the macrophages promotes a M2-like polarity. We also demonstrated that targeting PERK elevated glucose uptake in macrophages regardless of LPS stimulation, suggesting that the increased macrophage proliferative capacity (Figure 3B) and the elevated M1-like polarity markers (Figure 3A and 3D) may be a result of a metabolic shift. Moreover, we investigated the effect of UPR targeting on mitochondrial bioenergetics in RAW 264.7 macrophages without LPS stimulation using a Seahorse bioanalyzer (Figure 3D). IRE1 and PERK inhibition increased OCR and ECR suggesting an M1-macrophage polarity, while control transfected and GRP78-transfected cells had low OCR and ECR rates indicative of M2-like polarity. Therefore taken together these data suggest that GRP78 knockdown promotes lipid accumulation and ATGL activity to promote M2-like macrophage polarity, IRE1 knockdown stimulates both lipid accumulation and glycolysis resulting in a mixed population of M1/M2 macrophages, and targeting PERK elevated glucose uptake and enhanced glycolysis to favor a M1-like antitumor macrophage population (Figure 4D).

We isolated CD11b+ cells from the bone marrow of wild-type and GRP78 heterozygous mice. We showed that bone marrow-derived cells from GRP78 heterozygous mice display less M1-like macrophage markers (Figure 4A) and reduced cytolytic capacity (Figure 4C) when compared with wild-type bone marrow derived cells. We also observed that GRP78 heterozygousity prevented LPS-mediated reduction of UPR signaling components (Figure 4B). Taken together, these data indicate that inhibiting GRP78 in macrophages promotes an M2-like (pro-tumorigenic) phenotype possibly through the upregulation of other UPR signaling components and regulation of metabolism.

We previously observed that *in vivo* inhibition of tumoral GRP78 increased breast tumor infiltrating CD68 macrophages and was associated with increased therapeutic responsiveness [13]. In this model, GRP78 was inhibited only in tumor epithelial cells. These data suggest that GRP78 inhibition in cancer cells may regulate macrophage recruitment by modulating factors secreted from cancer epithelial cells. Indeed, inhibition of GRP78 significantly increased circulating MCP-1 concentrations [13]. We knocked down each UPR signaling arm individually (IRE1, GRP78, and PERK) in the 4T1B murine breast cancer cell line to determine the impact of UPR targeting in cancer epithelial cells on macrophage plasticity and cytolytic activity. We did not target ATF6 due to the redundant nature of ATF6 signaling; ATF6 promotes transcription of GRP78 and XBP1 to feed-back into UPR signaling. PERK inhibition in cancer epithelial cells had no effect on macrophage cytolytic activity. Cancer epithelial cell inhibition of IRE1 and GRP78 increased macrophage-mediated breast cancer cell death (Figure 5A). GRP78 was previously shown to localize to the cell membrane in cancer cells to promote PI3K and modulate Cripto-mediated TGF-β activation [37, 38]. Targeting GRP78 in cancer epithelial cells would also reduce cell surface GRP78, preventing anti-proliferative TGFβ signaling. However, TGF-β signaling is pleotropic; TGF-β also promotes tumor escape from immune surveillance, thereby suggesting that targeting cell surface GRP78 may modulate the anti-tumor immune response. Our group previously showed that targeting GRP78 in breast cancer cells and tumors reduced the “don't eat me” signaling protein CD47 levels [13]. CD47 is a widely expressed cell surface receptor that engages SIRPα on macrophages to inhibit phagocytosis [39, 40]. Reducing cell surface CD47 expression promotes macrophage recognition and phagocytosis, suggesting that targeting tumor epithelial GRP78 is a necessary component to promote an anti-tumor immune response.

Single cell type inhibition of UPR signaling components enabled elucidation of the particular contribution of each cell type response to the overall cell fate decision. A more translationally relevant model would include inhibiting UPR signaling components in both macrophages and cancer epithelial cells concurrently, simulating a systematic therapeutic treatment. Inhibition of each UPR signaling arm in both target (breast cancer) and effector (macrophage) cells indicates that systematic GRP78 or PERK targeting drugs may enhanced macrophage-mediated cancer cell death (Figure 6C). *In vivo* targeting of GRP78 in tumor bearing female BALB/c mice demonstrated elevated M1-like IL-12p70, IL-1, and IL-6 circulating cytokines with a corresponding decrease in M2-like TARC, eotaxin, and RANTES chemokines (Figure 6A), suggesting that systematic inhibition of GRP78 leads to a pro-inflammatory M1-like cytokine shift [41]. Furthermore, staining wild-type or GRP78 heterozygous breast tumors with CD68 (green) and CD80 (red) indicates that GRP78 heterozygousity increases the CD68+/CD80+ double positive cell infiltration into the breast tumor (Figure 6B), suggesting an elevated tumoral M1-like macrophage population. GRP78 heterozygous tumors also display a higher level of CD80 single positive cells, indicating an elevated monocyte/activated B-cells infiltrating the tumor. CD80 works in tandem with CD86 and is a critical component for T-cell priming [42–44]. These data suggest that targeting UPR may enhance the therapeutic potential of checkpoint inhibitors in immunotherapy, such as ipilimumab.

Ipilimumab is an immune checkpoint inhibitor targeting CTLA-4. Ipilimumab showed increased survival of patients with metastatic melanoma. While these immune checkpoint inhibitors have generated enthusiasm in the treatment of melanoma and other cancers, most patients do not respond. Acquired resistance was also manifested [45, 46]. These data highlight the need to understand the mechanism of immune checkpoint therapy resistance that occurs leading to melanoma disease progression [45]. Recently, it was demonstrated that macrophage polarity can influence resistance to immune checkpoint therapy by producing cytokines that result in inhibition of T cell activation and the production of cytokines that affect antigen presentation, causing overall suppression of T cell responses [19]. Moreover, in subsets of tumor of hepatocellular carcinoma patients, pro-inflammatory macrophages expressed PDL1 (B7-H1) which can engage T cells to cause suppression and immune tolerance in the tumor microenvironment [47]. Therefore, it was suggested that therapies which target macrophage polarization may enhance efficacy or increase responses to checkpoint inhibitor therapy [48]. Using matched pairs of human patient-derived PBMC obtained from melanoma patients before treatment and after disease progression on ipilimumab therapy, we identified a possible novel mechanism of immune checkpoint therapy resistance: regulation of UPR in the innate immune system. PBMC from patients had a decrease in the anti-tumor M1-like macrophages population with a corresponding increase in PERK and IRE protein expression observed after disease progression on ipilimumab treatment (Figure 7A and 7B). PBMCs obtained from patients after ipilimumab therapy resistance had an increase in CD206+ cells, demonstrating an elevated M2-like pro-tumorigenic macrophage population (Figure 7C). Furthermore, these CD206+ M2-like macrophages had increased PERK immunoreactivity, suggesting that UPR stimulation promotes M2 macrophage plasticity. These data suggest that ipilimumab activation of UPR signaling promotes a macrophage polarity shift promoting a pro-tumor M2-macrophage response, which may lead to ipilimumab therapy resistance. Taken together, these data suggest that UPR pathway inhibitors may enhance or improve effectiveness of immune checkpoint therapy blockade in melanoma patients. Overall, these studies demonstrate a novel role for UPR signaling activation in promoting an immunosuppressive tumor microenvironment, suggesting that targeting UPR may enhance the efficacy of immune checkpoint therapy in the clinic.

## MATERIALS AND METHODS

### Materials

The following materials were obtained as indicated: Mouse specific GRP78 (*HSPA5*), IRE1 (*ERN1*), and PERK (*EIF2AK3*) siRNA (Origene, Rockville, MD). ePlates were purchased from ACEA. RPMI media was purchased from Gibco Invitrogen BRL (Carlsbad, CA). Lipopolysaccharide (LPS), tunicamycin (Tn), and dithiothreitol (DTT) were from (Sigma-Aldrich). Antibodies were obtained from the following sources: GRP78, IRE1, PERK, iNOS, Arg-1, ATGL, and β-actin (Cell Signaling); IL12-Cy3, IL10-Cy7, CD68-FITC, CD80-Cy5, CD206-Cy7 (BioLegend, San Diego, CA). A CD11b positive cell isolation kit was obtained from eBioSciences. Animal experimentation was approved by the Animal Care and Use Committee of the Wake Forest School of Medicine (protocol # A16-010) and all methods were carried out in accordance with relevant guidelines and regulations. GRP78 heterozygous mice were previously purchased from Jackson Laboratories and a colony was maintained by our laboratory.

### Cell culture

4T1B murine breast cancer cell line, ZR-75-1 human ER+ breast cancer cell line, and RAW 264.7 mouse macrophage cell line were grown in phenol-red containing RPMI media containing 10% fetal bovine serum (FBS) and defined as basal growth conditions. Cells were grown at 37°C in a humidified, 5% CO_2_:95% air atmosphere.

### Macrophage isolation

Bone marrow derived CD11b+ cells were derived from 129S wild-type or GRP78 heterozygous mice using a positive isolation kit (eBioSciences) and plated in RPMI overnight. Adherent cells were treated with 1 μg/mL IFNγ for 48 hours followed by 1 μg/mL LPS stimulation for 24 hours and protein and RNA isolated for Western blot or RT-PCR analysis. CD11b+ cells were derived from 129S wild-type or GRP78 heterozygous mice and plated in an RTCA-ACEA e-plate to determine the effect of GRP78 heterozygosity on proliferation and macrophage cytolytic activity.

### Macrophage cytolytic assay

RAW 264.7 murine macrophage cells were treated with vehicle, 1 mM DTT or 1 μg/mL Tn, or were transfected with control, PERK, IRE1, or GRP78 siRNA overnight, counted and plated with 1 × 10^4^ 4T1B (triple-negative, murine breast cancer cells) in a 1:5 ratio of macrophages to cancer cells for 72 hours in the presence of treated in the presence of 1 μg/mL LPS (to activate macrophages). Macrophage killing was then assessed using the RTCA-ACEA xCELLigence^®^ system by measuring electrical impedance.

### Western blot

4T1B or RAW 264.7 cells were solubilized by sonication in radioimmunoprecipitation assay (RIPA) buffer lysis buffer. Proteins were size fractionated by gel electrophoresis and transferred to a nitrocellulose membrane. Nonspecific binding was blocked by incubation for 30 minutes at room temperature with Tris-buffered saline containing 5% powdered milk and 1% Triton X-100. Membranes were incubated overnight at 4°C with primary antibodies, followed by incubation with polyclonal HRP-conjugated secondary antibodies (1:5000) for 1 hour at room temperature. Immunoreactive products were visualized by chemiluminescence (SuperSignal Femto West, Pierce Biotechnology) and quantified by densitometry using the Bio-Rad digital densitometry software. Western blots are shown in figures as cropped images.

### RT-PCR

RNA was extracted using Trizol by following the manufacturer's protocol. cDNA was synthesized from 5 μg of total RNA using Superscript first strand RT-PCR reagents as described by the manufacturer. qRT-PCR was then performed using the Taq-man kit with specific primers for the following genes: NOS2, ARG1, TGFB, IL6, IL10, 18S.

### Oil-red-O staining

RAW 264.7 cells were transfected with scrambled control, GRP78, IRE1, or PERK targeting siRNA for 24 hours. Cells were then fixed and stained with oil-red-o for one hour at room temperature. Cells were washed and counterstained with hemotoxylin. Oil-red-o staining was visualized at 40× magnification using the Mantra Quantitative Pathology Image System.

### Glucose uptake

RAW 264.7 cells were transfected with scrambled control, GRP78, IRE1, or PERK targeting siRNA for 24 hours. Cells were then treated with 50 μg/mL 2-NDGB in for 24 hours. Glucose uptake was measured by fluorescent at excitation/ emission wavelength of 465/540 nm.

### Seahorse bioenergetic flux assay

Raw 264.7 macrophages were transfected for 48 h and plated at a 120,000/well in seahorse microplates. Mitochondrial metabolism was measured as the O_2_ Consumption Rate (OCR) and Basal Glycolysis was measured as the Extracellular Acidification Rate (ECAR). OCR and ECAR were measured in XF media (non-buffered DMEM containing 2 mM Glutamine pH 7.4) under basal condition and in response to Oligomycin 1 μM, FCCP 1 μM + Rotenone/Antimycin A 1 μM with the XF-96 Extracellular Flux Analyzer (Agilent Technologies).

### Immunofluorescence

RAW 264.7 mouse macrophage cells were treated with LPS, ZR-75-1 breast cancer conditioned media, or GRP78-silenced ZR-75-1 conditioned media for 24 hour. Expression of CD80, CD206, CD68, IL-10, and IL-12 was identified using immunofluorescence and visualized using the Mantra Quantitative Pathology Image System.

### GRP78 inhibition *in vivo*

As previously described, 4-week old female BALB/c mice were injected every three days I.P. with 30 μM mouse specific GRP78 targeting morpholino for three weeks before sacrifice. Mouse serum was collected and snap frozen for cytokine analysis [13].

### Cytokine analysis

Cytokines were measured as previously described [49]. In brief, serum from BALB/c mice treated with saline or mouse-targeting GRP78 morpholino were collected at necropsy and immediately frozen. Quansys Biosciences Q-Plex Array kits were used to measure the following mouse cytokines and chemokines: IL-12p70, IL-1β, IL-6, TARC, Eotaxin, and RANTES.

### Mammary carcinogenesis model

Wild type and GRP78 heterozygous mice were treated with a single MPA injection followed by 4 × 1 weekly doses of 1 mg DMBA in peanut oil to induce mammary tumorigenesis [22]. Once tumors formed, mice were treated with 400 ppm tamoxifen citrate chow. Tumors were fixed in formalin and paraffin embedded. Tissues were stained for CD68 and CD80 to identify infiltrating tumoral M1 macrophage population.

### Human PBMC

Blood samples were collected from patients with metastatic melanoma according to a protocol approved by the Wake Forest University Institutional Review Boards. All subjects gave informed consent prior to inclusion in the study. Circulating PBMC was isolated from patients before ipilimumab treatment or after progression of disease (CTLA-4 antibody therapy resistance). PBMC were plated for 48 hours and adherent cell population collected in RIPA buffer. Western blot hybridization was used to determine levels of UPR proteins along with the macrophage polarity markers iNOS and Arg-1. Adherent PBMC were also stained with fluorescent antibodies against CD206 and PERK to identify M2 macrophage populations and UPR signaling activity before and after CTLA4 antibody therapy resistance.

### Statistics

Data are presented as the mean ± standard error of the mean (SEM). Statistical differences were evaluated by Student's *t* test or one way analysis of variance (ANOVA) followed by Bonferoni *post hoc* tests. Criterion for statistical significance was set at *p* < 0.05.

## Abbreviations

UPR: Unfolded protein response
EnR: Endoplasmic reticulum
ER: estrogen receptor
IRE1: Inositol requiring enzyme 1
PERK: PKR-like endoplasmic reticulum kinase
GRP78: Glucose regulated protein 78
ATF6: Activated transcription factor 6
PBMC: Peripheral blood mononuclear cells
LPS: Lipopolysaccharide
IPI: Ipilimumab
DTT: Dithiothreitol
Tn: Tunicamycin
Arg-1: Arginase 1
iNOS: Inducible nitric oxide synthase
TGF-β: Transforming growth factor beta
